# Improving HIV pre-exposure prophylaxis persistence among adolescent girls and young women: Insights from a mixed-methods evaluation of community, hybrid, and facility service delivery models in Namibia

**DOI:** 10.3389/frph.2022.1048702

**Published:** 2022-12-05

**Authors:** Gena Barnabee, Gillian O’Bryan, Lylie Ndeikemona, Idel Billah, Lukas Silas, Karie L. Morgan, Katherine Shulock, Susan Mawire, Ellen MacLachlan, Josua Nghipangelwa, Elizabeth Muremi, Alison Ensminger, Norbert Forster, Gabrielle O’Malley

**Affiliations:** ^1^ International Training and Education Center for Health, Department of Global Health, University of Washington, Seattle, WA, United States; ^2^ Directorate of Special Programmes, Ministry of Health and Social Services, Windhoek, Namibia; ^3^ International Training and Education Center for Health, Department of Global Health, University of Washington, Windhoek, Namibia; ^4^ Center for Data Management and Translational Research, Michigan Public Health Institute, Okemos, MI, United States; ^5^ Disease Control and Health Statistics (DCHS), Washington State Department of Health, Seattle, WA, United States; ^6^ Star for Life, Windhoek, Namibia; ^7^ Oshikoto Regional Directorate, Ministry of Health and Social Services, Omuthiya, Namibia; ^8^ Khomas Regional Directorate, Ministry of Health and Social Services, Windhoek, Namibia

**Keywords:** delivery of health care, pre-exposure prophylaxis, HIV prevention, adolescent girls and young women, Namibia

## Abstract

**Introduction:**

Despite the potential for community-based approaches to increase access to pre-exposure prophylaxis (PrEP) for adolescent girls and young women (AGYW), there is limited evidence of whether and how they improve PrEP persistence. We compared PrEP persistence among AGYW receiving services through community and hybrid models in Namibia to facility-based services. We subsequently identify potential mechanisms to explain how and why community and hybrid models achieved (or not) improved persistence to inform further service delivery innovation.

**Methods:**

Data were collected from PrEP service delivery to AGYW over two-years in Namibia's Khomas Region. We used Kaplan-Meier analysis to estimate survival curves for PrEP persistence beyond three-months after initiation and report the cumulative probability of persistence at one- and three-months. Persistence was defined as any PrEP use within three months after initiation followed by a PrEP refill or previously prescribed supply of at least 30 days at the three-month visit. Interviews were conducted with 28 AGYW and 19 providers and analyzed using a deductive-inductive thematic approach.

**Results:**

From October 2017 through September 2019, 372 (18.7%) AGYW received services through a facility model, 302 (15.1%) through a community model, and 1,320 (66.2%) through a hybrid model. PrEP persistence at one- and three-months was 41.2% and 34.9% in the community model and 6.2% and 4.8% in the hybrid model compared to 36.8% and 26.7% in the facility model. Within the community and hybrid models, we identified three potential mechanisms related to PrEP persistence. Individualized service delivery offered *convenience and simplicity* which enabled AGYW to overcome barriers to obtaining refills but did not work as well for highly mobile AGYW. Consistent interactions and shared experiences fostered *social connectedness with providers and with peers*, building social networks and support systems for PrEP use. PrEP and HIV-related stigma, however, was widely experienced outside of these networks. Community-to-facility referral for PrEP refill triggered *apprehension towards unfamiliar PrEP services and providers* in AGYW, which discouraged persistence.

**Conclusion:**

Service delivery approaches that offer convenience and simplicity and foster social connectedness may reduce access barriers and increase social support enabling AGYW to self-manage their PrEP use and achieve improved PrEP persistence.

## 1. Introduction

Worldwide, adolescent girls and young women (AGYW) face disproportionate HIV incidence rates. In sub-Saharan Africa, AGYW account for one in four of all new HIV infections and close to four in five new HIV infections among young people (1, 2). Biological, structural, and socio-behavioral factors operate concurrently to increase AGYW risk of HIV acquisition and reduce their ability to utilize condoms and other prevention methods (2–6). Daily oral pre-exposure prophylaxis (PrEP) offers AGYW a discrete and user-led HIV prevention method. However, low PrEP persistence has been observed among AGYW across several studies and real-world delivery settings whether it has been defined as uninterrupted (i.e., continuous use from initiation to the time point at which persistence is assessed) or as persistence which allows for cyclical PrEP use (i.e., interruptions and subsequent restarts between initiation and the time point at which persistence is assessed) (7–13).

PrEP services are predominately offered at health facilities. However, AGYW may be hesitant to obtain PrEP from health facilities due to concerns about privacy and confidentiality, mistreatment by healthcare providers, long waiting times, costs associated with transport or services, and HIV-related stigma stemming from PrEP service delivery within facility HIV care and treatment clinics (14–18). For antiretroviral therapy, community-based and hybrid approaches to service delivery have been shown to improve patient outcomes (19). Community-based service delivery approaches may also be effective for PrEP, however there is limited evidence of *whether* such approaches improve PrEP outcomes such as persistence. Improving PrEP persistence is of particular importance to maximize PrEP's prevention effectiveness.

Effective use of community-based and hybrid models for PrEP service delivery to AGYW will also require a better understanding of *how* these approaches may work to achieve higher PrEP persistence and *why* they may work for some AGYW and not others. Inquiry into the mechanisms, or the ways in which any single or combination of service delivery components brings about change, may help answer these questions (20, 21). Better understanding of these mechanisms or “essential ingredients” can offer important insights to implementers and inform existing and future service delivery models and interventions to support improved PrEP persistence among AGYW.

In this mixed-methods paper, we explore *whether* and *how* PrEP service delivery through community and hybrid community-clinic models results in improved PrEP persistence among AGYW within the context of real-world PrEP service delivery to AGYW in the Khomas region of Namibia. We quantitatively assess PrEP persistence among AGYW receiving PrEP through community and hybrid community-clinic service delivery models and compare results to PrEP persistence among AGYW receiving PrEP through facility-based service delivery. We further use qualitative methods to identify potential mechanisms within the community and hybrid community-clinic models that contributed to or detracted from these approaches achieving improved PrEP persistence outcomes among AGYW.

## 2. Materials and methods

### 2.1. Study setting and design

We used mixed-methods to quantitatively assess whether and qualitatively explore how community and hybrid community-clinic models of PrEP service delivery improved PrEP persistence among AGYW compared to facility-based service delivery using routine data from PrEP service delivery in Namibia's Khomas Region from October 2017 through September 2019. Khomas has a highly urbanized population centered around the capital city of Windhoek (22). Within the region, UNAIDS estimates HIV incidence among AGYW as 3 to less than 10 infections per 1,000 uninfected population and 2 to 4 times higher than among their male peers (2).

Namibia first introduced PrEP as part of its 2016 National Guidelines on Antiretroviral Therapy. The Guidelines recommended PrEP be offered to any sexually active, HIV-negative person (including adolescents) who are at substantial risk of acquiring HIV (23). The following year, the National Strategic Framework for the HIV and AIDS Response in Namibia encouraged the strengthening of technical partnerships with domestic and international partner organizations as part of an ongoing learning process for PrEP scale-up (24). The DREAMS (Determined, Resilient, Empowered, AIDS-free, Mentored and Safe) program, a layered, multi-intervention approach to HIV prevention among AGYW, was launched in three regions of Namibia in 2018 (25). DREAMS provides participating AGYW aged 10-24 with age-appropriate HIV and gender-based violence (GBV) education, health services (including PrEP), social services, economic strengthening interventions and parent education (26). Interventions are delivered to small groups of AGYW at girls-only, community-based locations (“safe spaces”) in local community halls, schools, community organizations, and churches (27).

In Khomas, PrEP services were delivered *via* facility, community, and hybrid approaches. A “*facility model*”, where all PrEP services were delivered within public health facilities by public health providers was implemented in 10 public health facilities. A “*community-concierge model*” was comprised of fully community-based services delivered by DREAMS health providers. In this model, PrEP was initiated at DREAMS safe spaces and PrEP refills and follow-up services could be individualized for delivery at times and locations selected by the client, for example at a safe space, after hours at a secondary school, in or near their home, or at a market. Lastly, a “*hybrid community-clinic model*” consisted of community-based PrEP initiation at DREAMS safe spaces by DREAMS health providers and referral to the AGYW's preferred public health facility for PrEP refills and follow-up services by public health providers. Details of PrEP service delivery for each model are included in Supplementary Table S1.

PrEP services within public health facilities were implemented by the Namibian Ministry of Health and Social Services (MoHSS) with technical assistance provided by the International Training and Education Center for Health (I-TECH). AGYW could access facility-based PrEP services as walk-in clients or be referred while receiving other facility-based services such as HIV testing services, family planning, or antenatal care. The community-concierge and hybrid community-clinic models were implemented as part of the DREAMS and only available to AGYW participating in the program. DREAMS AGYW could choose their preferred model at PrEP initiation, however, the community-concierge model was only available during the last four months of the study period. During the study period, DREAMS in the Khomas region was implemented through a collaboration between MoHSS, the Ministry of Education, Arts and Culture, the Ministry of Gender Equality, Poverty Eradication and Child Welfare, I-TECH, Lifeline/Childline Namibia, and Star for Life. The program operated over 50 safe spaces primarily within Windhoek and the nearby surrounding area, where small groups of AGYW routinely received HIV and GBV education from “near” peer mentors (females aged 3–5 years older) and were visited by health and social service provider teams on agreed days and times.

PrEP services in all models were provided according to the latest Namibia National Guidelines available throughout implementation (23, 28). Medical officers and NIMART (nurse-initiated management of ART) trained nurses could provide PrEP. PrEP should be offered to any individual who tests HIV-negative, screens at substantial risk for HIV or considers themselves at risk, is without clinical contraindication, and is willing to regularly return to a service location for HIV testing, refills, and routine clinical visits. PrEP follow-up visits were recommended at one- and three-months, and every three-months thereafter. PrEP initiation should occur on the same day as screening and be offered as part of a combination prevention package that includes HIV testing services (HTS), male and female condoms, lubricants, antiretroviral therapy (ART) for partners living with HIV, voluntary medical male circumcision, and the prevention and management of sexually transmitted infections (STI). Adolescents should additionally be provided with sexual and reproductive health services including family planning counseling and methods.

### 2.2. Data collection and analysis

#### 2.2.1. Quantitative

Data were abstracted from paper-based MoHSS PrEP client records documenting client-level information at PrEP initiation (“baseline”) and at every subsequent PrEP refill or clinical visit. Baseline data included basic demographic characteristics, facility and service delivery characteristics, HIV risk factors and previous PrEP use. Information routinely documented at all PrEP visits included pregnancy status, symptomatic diagnosis of STIs, use and method of contraceptive, PrEP status, and PrEP regimen and quantity prescribed.

Descriptive analyses summarized demographic, PrEP service delivery, and clinical characteristics as well as HIV risk factors at baseline for all AGYW who initiated PrEP by service delivery model. Differences in characteristics across models were tested using Fisher's exact tests given the small sample sizes and adjusted for multiple testing. As persistence is a time-to-event variable, Kaplan-Meier survival analysis was used to evaluate PrEP persistence beyond three-months after PrEP initiation (29). Kaplan-Meier estimators and 95% confidence intervals (CI) were used to report the cumulative probability of PrEP persistence at one- and three-months after PrEP initiation by service delivery model. PrEP persistence beyond three-months was defined as any PrEP use (interrupted or uninterrupted) within three months after initiation followed by a PrEP refill or a previously prescribed PrEP supply of at least 30 days at the three-month visit (attended within 14 days of the scheduled follow-up date). Other studies have used similar definitions of persistence that allow for interruptions in PrEP use which may be appropriate given the cyclical nature of PrEP use and evidence which suggests AGYW, in the initial months of PrEP use, are still deciding whether PrEP is right for them (9, 18). Survival curves for each of the community and hybrid models were separately compared to the facility-based model using log-rank tests. Significance for all statistical tests was evaluated at *p* < 0.05. Analyses were conducted in RStudio (version 4.0.5) using the *survival* package (30, 31).

#### 2.2.2. Qualitative

From September to December 2019, in-depth interviews (IDI) were conducted with AGYW and healthcare providers (HCP). AGYW who started PrEP at health facilities and DREAMS safe spaces during the study period were purposively selected to obtain a mix of characteristics across age group (15–19 and 20–24) and service delivery model. HCP who provided PrEP services and were employed at health facilities or DREAMS safe spaces during the study period were purposively selected to obtain a mix of cadres involved in PrEP service delivery. Interviews were conducted by experienced qualitative interviewers using semi-structured interview guides (**S2: Qualitative Interview Guides**). The interview guide for AGYW explored individual experiences with PrEP and PrEP services, as well as factors influencing HIV risk perception and PrEP use decision-making. For HCPs, the interview guide explored provider experiences, attitudes, facilitators of, and barriers to delivering PrEP and PrEP services to AGYW. Interviews lasted between 30 and 90 min, were audio-recorded, and were conducted in-person and in English or, for AGYW, in the preferred local language. Recordings were simultaneously translated and transcribed into English. A second reviewer listened to all recordings and checked them against the transcripts, making revisions as necessary.

Transcripts were imported into ATLAS.ti (version 8) and analyzed using content analysis with higher-level abstraction and interpretation informed by theory-based evaluation concepts including mechanisms of change (20, 21, 32–34). The concept of mechanisms grew out of the realist evaluation approach which emphasizes understanding how interventions work in real world settings. Applied to models of PrEP service delivery, mechanisms are not the components or activities of service delivery itself, but rather the ways these influence the reasoning or response of AGYW, altering their behavior and contributing to a model's success or failure to improve PrEP persistence (20, 32, 34, 35). After reading through a subset of transcripts, two experienced qualitative researchers developed an initial code book used for both AGYW and provider IDIs to better identify and relate themes across datasets. The codebook included both inductive and deductive codes. A team of three researchers (KM, GO'B, KS) coded the transcripts through an iterative, collaborative coding and review process Analysis identified recurring themes and considered how mechanisms may have arose from the interactions between service delivery model components, activities, and context (36).

## 3. Results

### 3.1. PrEP persistence by service delivery model

From October 2017 through September 2019, 1994 AGYW aged 15–24 initiated PrEP with 372 (18.7%) receiving PrEP services through the facility model, 302 (15.1%) through the community-concierge model, and 1,320 (66.2%) through the hybrid community-clinic model (Table 1). Among AGYW receiving services through the facility model, 1,747 (55%) were aged 20–24, 74 (*n* = 74/368, 20.1%) were pregnant at initiation, 40 (*n* = 40/342, 11.7%) reported current use of oral or injectable contraceptives, and the most commonly reported HIV risk factors were having a partner living with HIV (*n* = 42, 11.3%) and having a partner with unknown HIV status (*n* = 155, 41.7%). In the community-concierge model, 116 (38.4%) of AGYW were aged 20–24, 4 (*n* = 4/301, 1.3%) were pregnant at initiation, 8 (*n* = 8/301, 2.7%) reported current use of oral or injectable contraceptives, none reported having a partner living with HIV, and 134 (44.4%) reported a partner with unknown HIV status. Among AGYW receiving services through the hybrid community-clinic model, 780 (59.1%) were aged 20–24, 11 (*n* = 11/1315, 0.8%) were pregnant at initiation, 58 (*n* = 58/1312, 4.4%) reported current use of oral or injectable contraceptives, 17 (1.3%) reported having a partner living with HIV, and 579 (43.9%) reported a partner with unknown HIV status. Across the three service delivery models, AGYW significantly differed by age group (*p* < 0.001), having a partner living with HIV (*p* < 0.001), inconsistent or no condom use (*p* = 0.002), having sex under the influence of alcohol and/or drugs (*p* = 0.029), previous PrEP use (*p* = 0.013), pregnant at initiation (*p* < 0.001), and being on oral or injectable contraceptives at initiation (*p* < 0.001) (Table 1).

**Table 1:** Characteristics of adolescent girls and young women who initiated PrEP (*n* = 1994) by service delivery model.

	Service delivery model			*p*-value^a^
	Facility	Community-concierge	Hybrid community-clinic	
Total, *N*	372	302	1320	–
Year PrEP initiated				–
2017	3 (0.8%)	0 (0%)	0 (0%)	
2018	205 (55.1%)	0 (0%)	210 (15.9%)	
2019	164 (44.1%)	302 (100%)	1,110 (84.1%)	
DREAMS participant	0 (0%)	302 (100%)	1,312 (99.4%)	–
**Demographic characteristics**				
Age	22 (20, 23)	19 (17, 21)	20 (18, 23)	**–**
Age group				**<0**.**001**
15–19	84 (22.6%)	186 (61.6%)	540 (40.9%)	
20–24	288 (77.4%)	116 (38.4%)	780 (59.1%)	
**HIV risk factors at PrEP initiation**				
Partner (s) living with HIV	42 (11.3%)	0 (0.0%)	17 (1.3%)	**<0**.**001**
Partner (s) HIV status unknown	155 (41.7%)	134 (44.4%)	579 (43.9%)	0.77
Inconsistent/no condom use	87 (23.4%)	84 (27.8%)	437 (33.1%)	**0**.**002**
Recurrent STIs	9 (2.4%)	2 (0.7%)	13 (1.0%)	0.11
Multiple concurrent partners	7 (1.9%)	7 (2.3%)	37 (2.8%)	0.77
Recurrent PEP use	2 (0.5%)	0 (0.0%)	2 (0.2%)	0.34
Sex under alcohol/drugs^b^	7 (1.9%)	1 (0.3%)	36 (2.7%)	**0**.**029**
Considers self at risk, only^c^	128 (34.4%)	140 (46.4%)	543 (41.1%)	**0**.**012**
**Clinical characteristics at PrEP initiation**				
Previous PrEP use	5 (1.3%)	4 (1.3%)	3 (0.2%)	**0**.**013**
Pregnant	74/368 (20.1%)	4/301 (1.3%)	11/1,315 (0.8%)	**<0**.**001**
Gestational age (*n* = 73)	22 (16, 28)	7 (4, 10)	16 (7, 24)	
On oral/injectable FP method	40/342 (11.7%)	8/301 (2.7%)	58/1,312 (4.4%)	**<0**.**001**
Symptomatic STI	5/355 (1.4%)	2/301 (0.7%)	14/1,299 (1.1%)	0.77
PrEP regimen prescribed				–
FTC/TDF	260/368 (70.7%)	60/301 (19.9%)	584/1,315 (44.4%)	
TDF/3TC	104/368 (28.3%)	240/301 (79.7%)	731/1,315 (55.6%)	
Other^c^	4/368 (1.1%)	1/301 (0.3%)	0/1,315 (0%)	

Data presented as *n* (%), median (IQR), or *n*/*N* (%) where data are missing. DREAMS, determined, resilient, empowered, AIDS-free, mentored, safe; FP, family planning; PEP, post-exposure prophylaxis; PrEP, pre-exposure prophylaxis; STI, sexually transmitted infection.

^a^ Fisher's exact tests with false discovery rate correction for multiple testing; significant values in bold.

^b^ Sex under the influence of alcohol and/or drugs.

^c^ Only HIV risk factor documented on the PrEP client record at PrEP initiation is “considers oneself at risk”.

**Figure 1:**
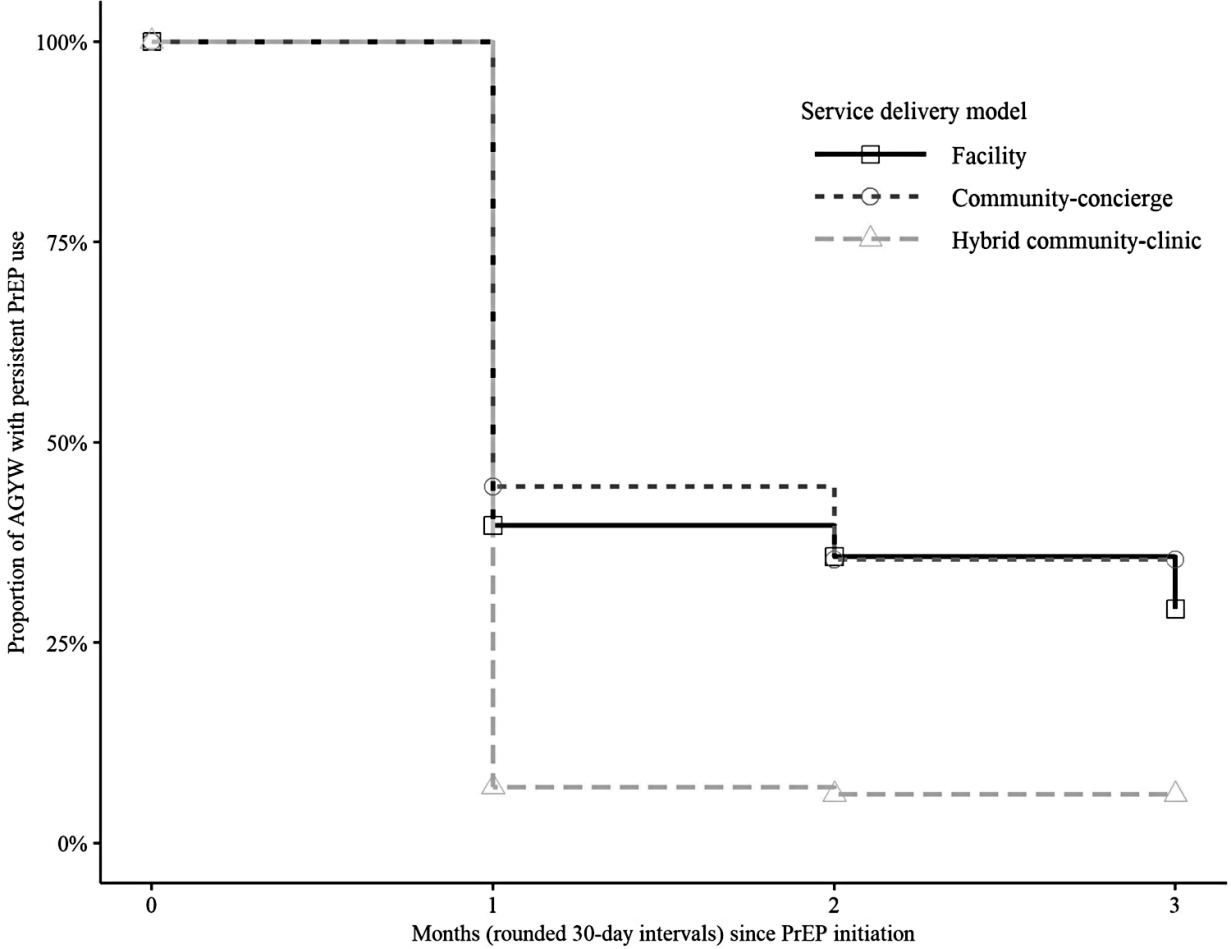
PrEP persistence (interrupted or uninterrupted) beyond three months among AGYW who initiated PrEP by service delivery model. Kaplan–Meier survival curves were used to estimate the cumulative probability of PrEP persistence beyond three months after PrEP initiation by service delivery model. AGYW who initiated PrEP during the study period (*N* = 1994) are included in the analysis. The marks on the curve indicate censoring and curves drop down when PrEP non-persistence (“failure”) events, defined as discontinuation of PrEP with no subsequent refill within three months after PrEP initiation, occur.

PrEP persistence at one-month after initiation was 36.8% (95% CI 32.1–42.2) in the facility model, 41.2% (95% CI 35.6–47.6) in the community-concierge model, and 6.2% (95% CI 5.1–7.7) in the hybrid community-clinic model (Figure 1). PrEP persistence at three-months after initiation was 26.7% (95% CI 22.4–31.9) in the facility model, 34.9% (95% CI 29.4–41.4) in the community-concierge model, and 4.8% (95% CI 3.8–6.2) in the hybrid community-clinic model. Differences in the survival curves for PrEP persistence beyond three months was non-significant between the facility and community-concierge models (*p* = 0.1) and significant between the facility and hybrid community-clinic models (*p* < 0.001).

### 3.2. Qualitative findings

We conducted a total of 47 IDIs, including 28 with AGYW who initiated PrEP and 19 with HCPs. Participant demographic characteristics are summarized in Table 2. Our qualitative inquiry focused on how the community and hybrid community-clinic models may have worked, or not, to improve PrEP persistence among AGYW. We identified two themes related to potential mechanisms supportive of improved PrEP persistence: *convenience and simplicity* and *social connectedness with providers and with peers*. We further identified one theme related to a detractive mechanism – *apprehension towards unfamiliar PrEP services and providers*. Illustrative quotations are included below and in Supplementary Table S3 and a synthesis of mechanism findings is included in Supplementary Table S4.

**Table 2:** Characteristics of in-depth interview participants.

Adolescent girls and young women	Healthcare providers
*n* (%)	(*N* = 28)	Median (IQR) or *n* (%)	(*N* = 19)
Age group		Age <35 years old	13 (68%)
15–19	12 (43%)	Female	16 (84%)
20–24	16 (57%)	Affiliated site	
DREAMS participant	24 (86%)	DREAMS safe space	7 (37%)
Service delivery model		Public health facility	12 (63%)
Facility	3 (11%)	Profession	
Community	10 (36%)	Nurse (RN/EN)	11 (59%)
Hybrid	15 (54%)	Health Assistant	6 (28%)
		Other^a^	2 (11%)
		Years in profession	8 (4.0, 10.5)
		Years delivering PrEP services at site^b^	1 (0.75, 2.0)
		Prior PrEP service delivery experience^b^	4 (36%)
		NIMART trained^c^	7 (70%)

^a^ “Other” includes pharmacy tech and DREAMS mentor.

^b^ Nurses only (*n* = 11).

^c^ Among PrEP prescribers (*n* = 10).

#### 3.2.1. Supportive mechanism: convenience and simplicity

The community-concierge model offered AGYW the ability to select the location and time of their PrEP refill and follow-up. Locations and dates were initially set at PrEP initiation, however, HCPs called AGYW individually in the week prior to their scheduled visit date to confirm or, where needed, change the visit location, date, and time. Using a phone number provided to them at initiation, AGYW could also contact providers to re-schedule, if needed.

*Basically, what we do is we call the clients and ask, “where do they feel comfortable with us seeing them?”… So we go wherever they feel it's okay for them. Some at home, some at school, some at work. When they are on lunch. Yes, wherever the client is. —HCP IDI 23, nurse*

AGYW explained that this made obtaining a PrEP refill convenient and simple. Benefits were often framed in opposition to the barriers typically faced when accessing PrEP in health facilities. For example, AGYW described how bringing the services to them meant they did not have to pay for transport and were less constrained by time as going to the clinic was often difficult with school, work, home, and other responsibilities. AGYW also described how an appointment confirmation call served as an important reminder and how if a refill visit was missed it was easy and quick to arrange for the provider to bring PrEP to them.

*Even if you had a follow-up to go to the clinic, sometimes they might not go anymore because by the time they come back from school, its late, they're tired, they might have things to do in the house, and the time is never enough. But if they bring the service to the high schools, even by the time a person knocks off before going to study or what, she can go get her pills. It's something really easy and nice. It's more motivating than walking here and there. —AGYW IDI 80, age 22*

*Yah, they can always remind you that tomorrow you have a follow-up, where can we find you. So, they bring the PrEP to you. Yah, it's very helpful because if maybe they used to say you must go get [PrEP] at the clinic, there is not even a thought already of taking them. Yah, because it's very challenging. Sometimes you don't even have cab money to go to the hospital. Sometimes you just forget like the follow-up itself. —AGYW IDI 89, age 22*

The individualized service delivery approach, however, did not always work for AGYW. Some AGYW described situations where providers were not able to reach them within their available windows of time as well as the difficulty in obtaining PrEP refills when traveling, which AGYW often cited as the reason for interruptions in PrEP use.

*We went to the village for a month because the holiday is taking a month. As I went there, I think just the second week at the village my follow date comes and I feel, I was trying to go to clinic and clinic is bit far. I just say, now I will not go. I will just continue [again] when [I] go [back] to school. —AGYW IDI 88, age 20*

The community-concierge model was resource-intensive with HCPs needing to provide individualized services to many AGYW, make multiple phone calls, arrange transport, and navigate unfamiliar areas of town. Implementation was facilitated by DREAMS employing dedicated staff for the coordination and delivery of PrEP refills as well as the provision of phone credit and transportation – which came in the form of dedicated cars and drivers, a mobile van that was outfitted for health service delivery, and mileage reimbursement if providers had to occasionally use their own vehicles.

*It's time, and the direction. Some don't know how to direct you; you will be just up and down… We spend two hours looking for the place, especially the place where there is no number, there is nothing. She will just direct you. We go [into neighborhoods] deep, deep there, down far, far. There is no number, there is nothing, so you have to try your best. —HCP IDI 24, health assistant*

Providers also noted several challenges of relying on mobile phone communication. For example, AGYW sometimes gave the number of their parent or partner which made it difficult to reliably and confidentially contact the AGYW. Providers also noted phone calls to AGYW did not always go through because their phones were switched off or lost, the number was switched, or because there was a lack of electricity to charge the phone.

*Calling the number, the number is off. Going to see someone, you wait even for one hour, even just waiting there outside in the sun and the person is not coming… Sometimes you might think a person has switched off the phone, maybe ignoring, doesn't want. But [actually] their battery is off because they’re staying far where there is no electricity. —HCP IDI 23, nurse*

Providers also noted the importance of and challenges to maintaining privacy when providing services to AGYW in the community. Practices implemented to maintain privacy in community-based service delivery included not wearing nurse uniforms and meeting in locations where AGYW felt safe and protected from view.

*You have to drive to a secret place. Where the neighbor can't see … what's going on. Some of them tell you “now you can come inside home.” “Cause those neighbors like to ask what happened, on the outside our nurses don’t wear uniform. Yah, it's sometimes if you say, ‘where is who-who?’ Then [she] says, ‘no she is not home,’ [because] she [doesn’t] want to be seen [with] the nurses going inside. People like to ask, ‘what happened I saw the nurse inside?". —HCP IDI 24, health assistant*

#### 3.2.2. Supportive mechanism: social connectedness with providers and with peers

Several components of the community service delivery approach fostered social connectedness between AGYW and providers as well as between AGYW and their peers. AGYW valued these connections which formed social networks from which they sought and received several types of support for PrEP use.

##### 3.2.2.1. Client-provider connectedness and support

Community-based PrEP service delivery approaches employed intentional strategies to foster positive socio-emotional relationships between service providers, including mentors and healthcare providers, and their AGYW clients. Strategies included the recruitment of “near” peers as mentors who provided HIV prevention and PrEP education, recruitment of young, female healthcare providers relatable to AGYW for the provision of HTS and PrEP services, emphasizing and guaranteeing confidentiality, and non-judgmental counseling and discussions with AGYW regarding their sexual relationships and behaviors. Many providers also employed additional strategies such as openly discussing their own experiences with HIV prevention and PrEP use and phoning clients shortly after PrEP initiation with the intention to check on the client's wellbeing rather than to provide reminders or tell them what to do. Providers perceived that building such relationships would build trust and allay common concerns and fears, such as judgement from providers and lack of confidentiality, which often lead AGYW to avoid seeking health services at facilities.

*Okay, we actually established a relationship with them where we assure them that whatever they talk about with us, it strictly stays between us. We are their big sister. We are not going to judge them. —HCP IDI 28, DREAMS mentor*

AGYW formed connections with providers and often described them as like an older sister or friend.

*They encouraged me that, “you don’t have to be afraid, be free.” If you have any problem go back to them, to go to them to the office to see how they can solve that problem… We were like friends, it's like we already knew each other (laughs). —AGYW IDI 75, age 24*

Antecedents to this feeling of connectedness included consistent interactions with the same providers, providers' friendly, empathetic, and non-judgmental approach, the perception that providers had similar experiences and faced similar challenges vis-à-vis their sexual relationships, the perception that providers trusted AGYW to make their own decisions about PrEP use, and the extra efforts made by providers to make them feel respected and cared for. This connectedness with providers enabled AGYW to discuss their relationships, HIV, and other challenges comfortably and openly with providers.

*‘Cause what we talk about in there, in the DREAMS program, remains there. It doesn’t go outside. And, yah, that's why I decided to open with them because I trust them. —AGYW IDI 94, age 18*

Trust in providers also had a spillover effect to PrEP. AGYW expressed confidence in the availability and accuracy of the information they were given about PrEP, that providers were not hiding information, and that providers were acting in the interest of the AGYW. This provided legitimacy to PrEP as well as a social support with the provider serving as an important source of emotional, informational, and appraisal support enabling AGYW to initiate and continue using PrEP.

*I like the way people at I-TECH/DREAMS treated us. They treated us good. And they motivated us… They even told us if we have questions, we should just ask them, they are free to answer our questions. —AGYW IDI 79, age 23*

*If I want to learn more about PrEP, I will consult a nurse. And I will ask them about it. Especially now the nurses that we have here at DREAMS program. I trust them, so I can always go ask them.* —*AGYW IDI 94, age 18*

AGYW expressed the importance of the information provided for using PrEP and making decisions about PrEP. AGYW described wanting essential, practical information such as about side effects, how to discontinue and restart PrEP, and what to do if a dose of PrEP is missed. AGYW also described situations where information on PrEP empowered them to persist when faced with challenges such as side effects or discouragement and stigma from others. Information, education, and communication (IEC) materials were also used to explain PrEP to parents or other influencers or gatekeepers of AGYW PrEP use.

*The first day I told them [my parents] when we were in DREAMS, it's like they didn't understand. Then I kept telling them every day we had DREAMS, telling more about it [PrEP]. Then I brought them a leaflet to read. Then they said, “that it's fine, it's your life, it's your decision. You have your own right. You can take it; we won’t stop you.” —AGYW IDI 74, age 22*

*The day I started taking PrEP, the next morning I started to feel like that [dizziness, vomiting, diarrhea] … because the nurse already told me that we have side effects… Yeah, because I was told that there would be side effects when I take PrEP. So when I decided to take PrEP, I knew there were side effects and I decided to take PrEP. — AGYW IDI 72, age 17*

##### 3.2.2.2. Peer-to-peer connectedness and support

Fostering connections between AGYW and their same sex and age peers was also an integral part of the service delivery in the community-based approach. In DREAMS, AGYW received HIV prevention education in small groups typically over the course of several weeks or months. Health services, including PrEP, were also provided to these same small groups. Mentors and providers also utilized group-based approaches for PrEP education and counseling.

These consistent interactions and shared experiences with other AGYW built and reinforced peer social networks which encouraged more in-depth discussions about PrEP. In groups, AGYW could build off each other's questions and ideas, and AGYW less comfortable with asking questions could benefit from those more open.

*Like you are in group and talk about it [PrEP] and talk more deeply, like of people who are in a group and talk about ideas to help others. People in a group always have different ideas. —AGYW IDI 88, age 20*

*Because sometimes there's something that touched you but it's not that serious, you can ask the mentor together with others listening, so that what you want to hear, there's probably somebody else that also wants to hear it. —AGYW IDI 80, age 22*

These interactions opened the door for AGYW to openly discuss PrEP with their peers and for some to disclose their PrEP use to their peers. Knowing other AGYW who took PrEP served as a powerful motivator for AGYW to continue with their own PrEP use.

*There were still people who were taking PrEP and people I knew, friends of mine took PrEP. So, I thought, like if they take, then I can even continue with my PrEP. —AGYW IDI 72, age 17*

Some AGYW also described peers as providers of adherence support whereby groups of AGYW who had all received community-based PrEP services would check in with each other to make sure they all took their pills or all friends would choose the same time to take their pill. Some AGYW intentionally provided support to their peers to continue with PrEP, often providing additional PrEP information or encouragement when AGYW were discouraged from using PrEP by people in their community.

*Yeah, so we would always talk about it. And she would always be like, we should always ask each other: did you take the pill today? And she would always take in the evenings. —AGYW IDI 73, age 15*

Some providers, however, expressed concern that AGYW based their decisions to take PrEP on information from their peers rather than on their own situation and level of HIV risk.

*Sometimes they just come like in a group. You went to the school or to the community, you find them in a group and then you talk to them. If a person is first saying, “Me I want [PrEP].” Then everyone is saying, “Okay, me I want [PrEP].” Cause the friend is also doing it. But then she did not come to her own decision, just doing it cause someone else is doing it. But in time, when she comes for follow-up is when she says, “Uh, uh (no), let me stop, that was a mistake, I was just taking something which I don’t know.” —HCP IDI 31, health assistant*

Importantly, peer interaction vis-à-vis PrEP did not always result in positive peer influence and support, particularly among friends who were not participating in DREAMS and not taking PrEP. Accurate knowledge of PrEP among these peers was low, leading to misconceptions about PrEP and, ultimately, remarks discouraging PrEP use.

*I had friends that were telling me, … “No you shouldn’t take PrEP, people that take PrEP are only those that have boyfriends … if you take PrEP [that means] you also have one.” —AGYW IDI 76, age 18*

*I always tell them [friends] … about PrEP and they always think that it's a joke. Like, “those things won’t work.” Those negative things. I just thought if you don't believe then that's [your] problem. …[T]hey are saying, “no, those things they don’t work, not 100%.”… It made me feel bad because I was trying to tell them, to educate them to take it, and they are just bringing me down saying, “no it doesn’t work,” and stuff like that. Okay, they said it's not 100%, the nurses told me that also, that I knew already, but them saying that it doesn't work without trying it, it doesn't make sense. —AGYW IDI 87, age 18*

#### 3.2.3. Detractive mechanism: apprehension over unfamiliar services and providers

AGYW expressed a clear preference for service delivery and provider continuity across PrEP initiation and refills and an averseness to obtaining PrEP through different modalities of service delivery and different service providers. This was strongly reflected in the experiences of AGYW who received services in the hybrid community-clinic model where referral from community services to facility services for PrEP refills triggered apprehension in AGYW over unfamiliar PrEP services and interacting with unfamiliar providers which, for some, led to an interruption in PrEP use.

*I was thinking that maybe when I go visit the clinic maybe I will not find the [community] nurses there or maybe going to my follow-up, I will not know where to go. —AGYW IDI 96, age 16*

Those who did seek PrEP refills at the facility after community-based PrEP initiation often described encountering challenges in navigating unfamiliar services. For example, AGYW described unexpected costs and confusion about where to go, sometimes needing to pass by multiple service delivery points or waiting in one queue only to eventually be sent back to a different one altogether.

*Ok, the first follow up I had was here in Windhoek at [the clinic]…[T]hat was my first time going to that clinic. So, I stood in a long line…and we had to go back, people were just sending me back. So that took almost the whole day for me to get the PrEP and I also had to pay 8 dollars for the follow up. Which was confusing…. —AGYW IDI 87, age 18*

In seeking PrEP refills from unfamiliar providers, AGYW expressed feeling frustrated and discouraged at being repeatedly asked to explain and justify their reason for taking PrEP, sometimes at multiple delivery points or by multiple providers in a single visit. AGYW also described feeling less comfortable with unfamiliar providers, particularly when providers were unfriendly or unsupportive. Others described mixed messages they received from providers within different service modalities. For example, community providers would say they could use PrEP whereas some facility providers would say that PrEP was only for people with partners living with HIV or that PrEP was not for adolescents, and sometimes denied their request for a PrEP refill.

*When I went there at first at the clinic, because that time the pills that I got from [the community] it was finished. So I went there, then they said, “what are you looking for here?” Then I said, “I came to get my PrEP.” Then they said, “you should go inside the clinic, then you go for HIV testing”… When I went there, the woman asked me, “why am I there?” Then I told her that, “I’m on PrEP.' Then she asked me, “at your age?”… She tested me, then she said I should go outside and wait a bit… When I went in the pharmacy ….[he] was asking me again, “why are you taking PrEP?” Then I was just quiet. Then he said, “if you are not going to answer me then I’m not going to give your pills because you are just drinking something that you don't know what is it.” Then I said, “I’m taking PrEP because I don't want to get HIV.' Then he said, “why… are you not like having one partner?’ Then I said, “I’m having.” “And is your partner having HIV?” Then I said no, then he said, “oh, so why are you taking it? PrEP is for only people that they are in a relationship whereby one partner is [living with] HIV.” Then I said, “you never know, maybe your partner is lying to you.”… Then he said ok, then he gave me, then I went home. —AGYW IDI 78, age 24*

Having already been taking PrEP, AGYW expected that it would be easy to get refills. Instead, these challenges made it harder for AGYW to get their PrEP refill and, for some, reduced their motivation to return to the clinic for future refills or to even take PrEP at all.

*A [provider] comes by, you aren’t used to her. It's the first time but you are just [being mistreated]…You might just find yourself giving up, not coming back. You might find yourself stopping to take your medicine. —AGYW IDI 71, age 18*

## 4. Discussion

We quantitatively assessed the cumulative probability of PrEP persistence beyond three-months after initiation among AGYW receiving services through community-concierge and hybrid community-clinic models and compared results to persistence among AGYW receiving PrEP *via* facility-based services. PrEP persistence was moderate in the community-concierge model and low in the hybrid community-clinic model. The community-concierge model achieved higher persistence compared to facility-based service delivery, however the difference was not statistically significant. The hybrid community-clinic model, on the other hand, achieved substantially and significantly lower persistence. Through qualitative analysis, we further identified mechanisms within the community and hybrid models which supported or detracted from persistent PrEP use. Supportive mechanisms included convenience and simplicity as well as social connectedness with providers and with peers. Detractive to persistence was apprehension over unfamiliar PrEP services and providers, which was strongly reflected in the experiences of AGYW receiving services through the hybrid community-clinic model.

Given that the community-concierge model was designed to address many of the barriers to PrEP access and use which have been previously identified in the literature, an unexpected finding in our study was its failure to achieve significantly improved PrEP persistence compared to facility-based service delivery. We propose a few hypotheses related to compound factors or influences as to why this occurred. First, consistent with other research we found PrEP stigma perpetrated by peers and family members as well as high geographic mobility to be key contributors to interrupted PrEP use among AGYW across all service delivery approaches in this study (18, 37). It is possible that these factors, particularly when they occur early in PrEP use, impose a greater influence on persistence than the supportive mechanisms identified in this study. Additionally, AGYW receiving services through the different service delivery models in our study might have fundamentally differed, as we imagine is the case in real-world delivery contexts. For example, those initiating PrEP at health facilities may be those who have already demonstrated their willingness and ability to access facility-based services and may not experience the same barriers presented by facility-based service delivery as other AGYW. In addition, higher proportions of the AGYW receiving services through the facility model were aged 20–24, were pregnant at PrEP initiation, reported oral or injectable contraceptive use, or had reported one or more partners living with HIV, all factors shown to be associated with higher PrEP persistence in other studies (7, 9, 38, 39). Implementation time may have also affected persistence as the community-concierge model was only implemented in the last four months of the studied period and persistence may have continued to increase over time as improvements were made in response to identified challenges. Lastly, small sample sizes resulted in wide confidence intervals and may have reduced our ability to detect a significant difference between the models.

The World Health Organization (WHO) recommends differentiated approaches that simplify and decentralize PrEP service delivery (40). Our findings demonstrate that an individualized, “concierge” approach can bring PrEP refills and follow-up services to AGYW on days, at times, and to nearby locations convenient for them and simplify the process of scheduling refills and making changes to help AGYW overcome access barriers that often lead to interruptions in PrEP use. Results from the Delivery Optimization for Antiretroviral Therapy study similarly identified *flexibility* in terms of scheduling and re-scheduling refill appointments of community-based ART delivery to greatly reduce access barriers and improve service quality which led to improved rates of viral suppression among people living with HIV compared to facility-based services (19, 41). Individualized delivery of PrEP refills, however, is resource-intensive and its sustainability may require the use of triage tools to identify AGYW who could benefit most from this approach (42). Widespread and consistent availability of PrEP in community pharmacies or other community-based locations may also successfully offer convenience and simplicity without the resource burden associated with an individualized approach (43, 44).

Persistent PrEP use was also supported by social connectedness between AGYW and their providers and their peers, building and reinforcing social networks from which AGYW received emotional, informational, and instrumental support. Providers and peers also using PrEP may be critical sources of social support especially for AGYW who choose to conceal their PrEP use. Several studies have shown the importance of social support in the uptake and continued use of PrEP as well as contraception (8, 45–47). Peer-supported PrEP service delivery methods such as peer clubs have also been shown to increase PrEP persistence among AGYW (48). Peer-led service delivery models in Thailand and Namibia have successfully delivered PrEP refills to key populations, however, further research is needed to develop and scale peer-led PrEP service delivery models for AGYW (49–51).

In our study, apprehension over as well as actual negative experiences while seeking PrEP refills from unfamiliar services and providers may have largely contributed to the low rate of PrEP persistence observed among AGYW receiving services in the hybrid community-clinic model. Scaling PrEP service delivery, however, is likely to require hybrid approaches whether client-led – where AGYW seek and utilize different service delivery approaches based on their needs at different points in time – or program-led – where the availability of differentiated PrEP service delivery options are limited by financial, programmatic, and human resources (44). Programs could consider several implementation strategies to overcome this barrier. Centralized, accessible information on where AGYW can find PrEP services through mobile phone applications or websites could help AGYW prepare for and access services in different locations, particularly if they include *enough* information such as details on PrEP service flow within each location. B-wise, a mobile health information and communication platform used in South Africa, is one example that could be adapted and replicated in other settings (52). Where providers are well-informed about available PrEP service modalities, client-centered counseling could support AGYW to anticipate challenges in accessing PrEP services, help AGYW to determine which service delivery modalities may enable more persistent PrEP use, and provide practical information to familiarize them with service delivery (53). Peer-navigators could also be utilized to improve linkage of AGYW across different service modalities making AGYW feel welcomed and familiarizing AGYW with new services and providers.

The strength of our study lies in its assessment of PrEP persistence and identification of mechanisms supportive and detractive of persistent PrEP use among AGYW receiving services through real-world, programmatic service delivery approaches within and outside of health facilities. The study also has several limitations. We used PrEP service delivery and qualitative data collected from just one region in Namibia, which limits the generalizability of our findings to other delivery settings. Persistence may have been underestimated due to the limitations of programmatic data; reliance on paper-based records limited the ability to link a client's source record with any PrEP refills received at a different facility or community service provider. Due to the inability to link PrEP client and pharmacy records, we used PrEP prescription data to measure persistence, which may overestimate persistence in the facility and hybrid models where PrEP refill dispensing occurred at the pharmacy rather than in-room by the prescribing provider. While our study is strengthened by the inclusion of qualitative data from both AGYW and providers, the mechanisms identified are limited to the experiences of these participants and do not reflect the entirety of mechanisms operating on persistent PrEP use among AGYW.

## 5. Conclusions

Differentiated models of PrEP service delivery, particularly those operating outside of health facilities, are needed to increase PrEP access and use among AGYW. Delivery approaches which offer convenience and simplicity in accessing PrEP refills and foster social connectedness to build social networks and support systems between AGYW and their providers as well as with peers may improve PrEP persistence. Further research into additional strategies and mechanisms supportive of PrEP outcomes (i.e., uptake, and persistence) are needed to inform effective implementation of community-based and other innovative PrEP service delivery approaches for AGYW and, ultimately, for PrEP to reduce the substantial HIV burden among this population.

## Data Availability

The data included in this manuscript was sourced from various study collaborators. Data requests should be made to Gena Barnabee at genab@uw.edu and reasonable requests will be relayed to the appropriate collaborator.
