# Supplementary material for: Improving HIV pre-exposure prophylaxis persistence among adolescent girls and young women: Insights from a mixed-methods evaluation of community, hybrid, and facility service delivery models in Namibia
Source: Front Reprod Health. 2022 Dec 5;4:1048702. doi: 10.3389/frph.2022.1048702 (PMC9760915; doi:10.3389/frph.2022.1048702)
Supplement: Supplementary file 1 [file Table1.docx]

**Table S1**. Description of facility, community-concierge, and hybrid community-clinic service delivery models^1^

|  | Facility model | Community-concierge model | Hybrid community-clinic model |
| --- | --- | --- | --- |
| *Summary* | All services delivered in government health facilities by government healthcare providers | All services delivered in community-based locations by community-based providers | Initial PrEP services delivered in community-based locations by community-based providers with referral to client’s preferred government health facility for PrEP refill and clinical follow-up by government healthcare providers |
| *Population(s) served* | All populations | Only adolescent girls and young women | Only adolescent girls and young women |
| Initial PrEP services |  |  |  |
| *Location* | Government health facility | Community-based safe space | Same as community-concierge model |
| *Entry point(s)* | Self-referral or by provider referral if attending the facility for another service (e.g., ANC, FP, PHC) | All DREAMS participants aged 15-24 received PrEP education, HIV test, HIV risk screening, and PrEP offer (when indicated) | Same as community-concierge model |
| *Level of integration* | Facility-dependent; services most often not integrated | Location and provider-level integration; all services provided in same room with HTS and clinical consultation areas separated by privacy screens | Same as community-concierge model |
| *PrEP education  and initial counseling* | Provided in the HTS room by HTS provider  or in ART room/ART clinic by ART nurse | Education provided by the peer mentor during HIV/GBV prevention education session(s)  Education also provided in the waiting area on health services drop-in days at the safe space  Education and initial counseling provided by HTS provider after a negative HIV test result and/or by the nurse during the clinical consultation | Same as community-concierge model |
| *HIV counseling and testing* | Conducted in HTS room by HTS provider | Conducted in HTS area by HTS provider  or in the consultation area by the nurse | Same as community-concierge model |
| *HIV risk screening* | Conducted in HTS room by HTS provider  or in ART room/clinic by ART nurse | Conducted in consultation area by the nurse | Same as community-concierge model |
| *Clinical services^2^ and PrEP prescription* | Provided in ART room/ART clinic  by the ART nurse | Provided in consultation area by the nurse  Integrated with package of SRH services^7^ | Same as community-concierge model |
| *Adherence counseling^3^* | Provided in consultation room by ART nurse | Provided in consultation area by the nurse |  |
| *PrEP dispensation* | Dispensed at ART pharmacy (or general pharmacy if only one) by pharmacist/pharmacy assistant | Dispensed in the consultation area by the nurse | Same as community-concierge model |
| *Laboratory tests^4^* | As recommended by MoHSS guidelines  Bloods drawn in phlebotomy room by phlebotomist or trained nurse | As recommended by MoHSS guidelines  Bloods drawn in consultation area by nurse; samples dropped at nearest facility for same-day NIP pick-up | Same as community-concierge model |
| PrEP refill and follow-up services | | | |
| *Location* | Government health facility | Community-based safe space or other community-based location preferred by client | Same as facility model |
| *Level of integration* | Facility-dependent; services most often not integrated | Location and provider-level integration | Same as facility model |
| *Refills prescription and dispensation* | 1-month after initiation, every 1-3 months thereafter dependent on provider discretion (e.g., more frequent visits in response to stock-outs of PrEP medications or adherence concerns)  Dispensed in ART pharmacy (or general pharmacy if only one) by pharmacist/pharmacy assistant | 1-month after initiation, every 1-3 months thereafter dependent on client preference (e.g., to align with family planning method provision, travel plans, or other client preferences) or provider discretion (e.g., more frequent visits in response to stock-outs of PrEP medications or adherence concerns)  Dispensed in consultation area by nurse alongside family planning method when possible. | Same as facility model |
| *Clinical monitoring^5^* | 1-month and 3-months after initiation  and every 3 months thereafter  Provided in ART room/ART clinic  by the ART nurse  Services included HTS, PrEP/adherence counseling, and assessment and management of side effects | 1-month and 3-months after initiation  and every 3 months thereafter  Provided in consultation area by nurse as part of an integrated SRH service package.  Services included HTS, PrEP/adherence counseling, and assessment and management of side effects | Same as facility model |
| *Laboratory tests^6^* | As recommended by MoHSS guidelines  Bloods drawn in phlebotomy room by phlebotomist or trained nurse | As recommended by MoHSS guidelines  Bloods drawn in consultation area by nurse; samples dropped at nearest facility for same-day NIP pick-up | Same as facility model |
| Additional components | | | |
| *Additional psychosocial support* | None | Provided a phone number which AGYW could call or send an SMS or WhatsApp message with any questions or concerns and receive a response via text or phone call by a DREAMS nurse  All DREAMS participants received GBV screening and (if indicated) counseling and case management | Same as community-concierge model |
| *Refill / follow-up appointment reminders* | Some providers may have conducted reminder phone calls after missed refill/follow-up visits as this was routinely done for ART clients. | Phone call within 1 week prior to scheduled refill/follow-up date to confirm location, day, time  SMS or phone call reminders after missed refill/follow-up visits | None |
| ANC: antenatal care. ART: antiretroviral therapy. FP: family planning. HTS: HIV testing services. MoHSS: Ministry of Health and Social Services. PHC: primary healthcare. SRH: sexual and reproductive health. ^1^ Description of models as implemented in Namibia between October 2017 and September 2019 in Khomas region, Namibia. ^2^ Guidelines recommended: . ^3^ Guidelines recommended: . ^4^ Guidelines recommended: . ^5^ Guidelines recommended: . ^6^ Guidelines recommended: . ^7^ Included family planning counseling and method provision, symptomatic screening and treatment for sexually transmitted infections, and condoms provision. | | | |
