# Supplementary material for: Improving HIV pre-exposure prophylaxis persistence among adolescent girls and young women: Insights from a mixed-methods evaluation of community, hybrid, and facility service delivery models in Namibia
Source: Front Reprod Health. 2022 Dec 5;4:1048702. doi: 10.3389/frph.2022.1048702 (PMC9760915; doi:10.3389/frph.2022.1048702)
Supplement: Supplementary file 2 [file Table2.docx]

**Supplementary File 2: Qualitative Interview Guides:** In-depth interview guides for adolescent girls and young women and healthcare providers

**In-depth Interview Guide – Adolescent Girls and Young Women**

***Theme 1: Knowledge of PrEP [for ALL]***

First, I’d like to learn more about how you learned of PrEP.

1. How did you first hear about PrEP?
2. What is PrEP, according to your own knowledge?
   1. Who can take it? Why?
   2. Why might PrEP be helpful for young women like you?
   3. How are people supposed to take PrEP?
3. Are you interested in learning more about PrEP? Why/why not?

**[if yes]**

- 1. What would you want to find out?
  2. Who or where would you go to learn more about PrEP and why?
  3. Have you already tried to get more information (from whom, where)? How did that go?

***Theme 2: PrEP Decision [for ALL]***

Now, I would like you to think about when you first made your decision about whether or not to start PrEP. We want to learn more about how you made that decision, what things you thought about, and whether what others said or what you thought others might think affected your decision.

1. ***[if she is NOT a DREAMS participant]***
   1. On the day you started PrEP, why did you come to the clinic that day?
      1. **[If came to clinic only for PrEP]** What or who encouraged or motivated you to come?
2. How did you decide if you wanted to start PrEP?
   1. What were some of the things you considered when making your decision?
      1. Concerns or risk of getting HIV
      2. Concerns about your relationship/your sexual partner(s)
      3. Concerns about drinking PrEP (e.g., pill size, side effects, pregnancy)
      4. Concerns about stigma/discrimination
3. When you were making your decision whether or not to start PrEP, what possible difficulties did you think about?
   - 1. ***[If this question is not clear, offer examples:]*** Accessing PrEP, time spent at the clinic; privacy; ability/willingness to take daily; forgetting; pill storage, pill burden, HIV testing
4. How did people in your life influence your decision of whether or not to start PrEP?

(Explain that influence may be good or bad, it could be something someone said to you, or what you thought others might think or say).

- 1. Did you talk with anyone while making your decision?
     1. How did that go?
  2. Do you know anyone else using PrEP currently or who previously used PrEP?

1. Have you talked with them about their experiences using PrEP?
2. What did you hear from any healthcare worker or DREAMS staff (e.g., Girl Mentor, social worker) that helped you to decide whether or not to start PrEP?
   1. What did they tell you about PrEP?
   2. After talking with this person, what questions did you have about PrEP?
3. How did they address those questions?
   1. How comfortable were you talking about sex, HIV and PrEP with this person?
4. What are some ways healthcare workers or DREAMS staff can better help you or other young women like you decide whether or not to start PrEP?
5. Did you make your decision about starting PrEP at the clinic or later on at home?
   1. If at the clinic, what made you ready to decide at that time?
   2. If later on at home, what else did you need to think about or do in order to make your decision? Why?
      1. If you weren’t completely decided at the clinic/site, what made you take the pills home at the time?
6. **[If decided not to start PrEP]** Do you think you may start PrEP in the future?

Why/Why not?

- 1. Can you imagine a situation in which you may want to use PrEP in the future?
     1. **[if yes]** Can you tell me more about it?

***Theme 3: Experiences using PrEP* *[if she decided to start PrEP]***

Now I would like to talk about your experience using PrEP.

1. Tell me about how you take PrEP.
   1. When was the last time you drank a pill? What were you doing? Where were you?
   2. How do you choose when to drink or not to drink PrEP?
   3. Where have you been storing the pills? Any challenges with storage?
2. Did you tell anyone that you are using PrEP? Why/why not?
   1. How did that go?
3. What kinds of things have helped you or made it easy for you to take PrEP?

**(Probe on what has helped drink the pills every day and to continue over time)**

- 1. What strategies have you used to help you to take PrEP.
  2. What support have you received that has helped you take PrEP? (e.g., support groups, financial support)
  3. What or who has motivated or encouraged you and how?

**Probe specifically about person(s) mentioned in Q13 if the participant does not mention them.**

1. What kinds of things have made it difficult for you to take PrEP? How so?

**(Explain that this can also include people who have made it difficult)**

- 1. How have you handled these challenges?

1. How has taking PrEP made you feel?
2. How have you felt physically? (Any side effects? Any positive effects?)
3. How do you feel emotionally?
4. Do you feel confident? Scared?
5. Tell me about a time you missed drinking your PrEP pill.
6. What caused you to miss drinking your pill?
   - 1. What did you do when you realized you missed taking your pill?
7. Did you talk to anyone about it? Why or why not?
   - 1. **[if yes]** What did they say? Was it helpful? Did you make any changes?
8. What could help you to not miss drinking your pills?
9. Can you think of a time when you had many challenges, but you still were able to drink your pill?
10. **[if yes]** Tell me more about that time (what was the challenge, what helped).
11. Have you ever stopped PrEP for a while?

***[if NO]***

- 1. How long do you plan to continue to use PrEP?
  2. Are there any reasons what you would stop using PrEP? What are these?

***[if YES: if she stopped drinking PrEP for a while or completely, Q 20-22]***

1. What made you stop or decide not to drink your pills for a while?
2. Were there any reasons you didn’t think you needed or wanted to use PrEP anymore? Can you tell me more about this?
3. Did you learn or hear anything that made you change your mind about using PrEP? What information changed your mind? Where did you hear it?
4. How did people in your life influence your decision to stop using PrEP?
5. Were there any changes in your life (in your household, work, income, other) that made it harder or less important for you to use PrEP?
6. How did changes in your health affect using PrEP? (e.g., side effects, pregnancy)
7. Are there any other reasons that caused you to stop taking PrEP?
8. Did anything happen while receiving PrEP services that made you decide to stop using PrEP? Tell me more (e.g., attitude of the provider, challenge with getting services).
9. Have you restarted PrEP since stopping?

**[if yes]**

- 1. Why did you decide to start again?
  2. Did you experience any challenges or difficulties in starting again (e.g., scolded by healthcare worker)?

**[if no]**

1. Do you think you will use PrEP in the future? Why/Why not?
2. Can you imagine a situation in which you may want to use PrEP in the future? If yes, can you tell me more about that?

***Theme 4:*** ***Service Experience [for ALL]***

Next, I would like to learn more about experiences in receiving PrEP services.

1. Can you tell me about your PrEP services visit on the day you started PrEP?
   1. Where did you have to go? How did you know to go there?
   2. What happened during this visit?
   3. Was there anything that you didn’t like or that you found uncomfortable?
   4. How well did the counseling provided that day help prepare you to take PrEP?
      1. What else do you think should have been included? What was confusing? What was most useful or helpful?
   5. How were you treated by the healthcare workers who worked with you that day?
      1. Can you give me an example of how someone treated you well or badly?
2. Can you tell me about the first time you went for a follow-up visit?
   1. Where did you have to go?
   2. What happened?
3. Was there anything that made this feel easier or more difficult than before?
   1. How were you treated by healthcare workers?
4. How have other follow-ups been similar or different than this one?

1. Can you tell me about any support you received from healthcare workers/DREAMS outside of these visits?
   1. Did you ever contact a healthcare worker?
      1. Why did you contact him/her? How did that go?
   2. What other kind of support would you have liked to receive from healthcare providers/DREAMS providers or others?
2. How would you change PrEP services so it would be easier for you or other young women to start and continue PrEP (e.g., locations available, providers)?
   1. Can you give me some examples?
3. In your opinion, what do you/would you like about being able to get PrEP services somewhere in the community (e.g., outside of facilities)?
   1. What do you not like as much about it?
   2. Where in the community would be best? Why?
4. In your opinion as a young woman, what do you/would you like about being able to get PrEP services in a health facility?
   1. What do you not like as much about it?
   2. Where in a health facility would be best? Why?

**[If started in the community/DREAMS]**

1. If PrEP had not been available to you through DREAMS, would you have gone to a facility to start PrEP? Why/why not?

**[If started in the facility]**

1. If PrEP had been available to you somewhere in the community, would you have still started PrEP at the facility or chosen the community location? Why?

**[If follow-up done in the community/DREAMS]**

1. What did you like about getting PrEP follow up in the community?
   1. What did you not like as much about it?
   2. If PrEP follow-up was only available in a health facility, would you have gone to the facility for follow-up? Why/why not?

**[If started in community/DREAMS and follow-up done in facility]**

1. What did you like about getting PrEP follow up in a health facility?
   1. What did you not like as much about it?
   2. If PrEP follow-up was also available somewhere in the community, where would you chose to go for follow-up? Why/why not?

**[If no follow-up attended]**

1. Were you asked to go to a facility for follow-up?
   1. **[If yes]** How did that influence whether or not you attended your follow-up? If PrEP follow-up was also available somewhere in the community, would you have attended? Why?

***Theme 5:*** ***Relationships [for ALL]***

Next, I would like to learn more about your relationships with men.

1. Tell me about your sexual partner and what your relationship is like.

**(sexual partner can be boyfriend, ‘situationship’, serious or casual)**

- 1. What kinds of things are easy to talk about with this person? What kinds of things are hard to talk about?
  2. Do you have other sexual partners?

1. Tell me about your relationships with them.
2. In these relationship(s) or any you have had in the past, have you ever talked about HIV?

***[if yes]*** a. Tell me more about what you’ve talked about.

- - 1. What were the situations in which you discussed HIV?

***[if no]*** *b.*  Tell me more about why you haven’t talked about HIV?

- 1. What are the challenges in talking about HIV in your relationships?
  2. What could service providers, or others do, to help you overcome those challenges?

c. Have you ever had an HIV test together with your sexual partner(s)?

***[if yes]*** Do you know your current sexual partner(s)’ HIV status?

***[if no]*** Why do you think this hasn’t come up?

1. Have you told your sexual partner(s) that you are using PrEP or that you were thinking about using PrEP? Why/why not?
2. ***[if yes]*** And how did that go?
3. Can you tell me about how he reacted to your use of or interest in PrEP?
4. ***[if no]*** What made you decide to keep your use of PrEP a secret?

***Theme 6:*** ***Stigma and Discrimination [for ALL]***

Now, I would like to learn more about stigma and discrimination related to PrEP that you have experienced or that you have heard about in your community.

**[if she used PrEP at all]:**

1. Can you tell me about any positive or negative experiences you have had with other people because you are using PrEP?
   1. Who was it and what did they say or do?
      1. How is this related to you using PrEP?
   2. How do you feel about what they said/did?
      1. Do you think what they say is true or false? Why?

**[for ALL]:**

1. Now I would like to talk about what people in the community are saying about PrEP. Can you tell me about any things you have heard in your community about PrEP? ***[This is however she defines community – can be neighborhood, peers, region etc.]***
2. What kinds of ideas/rumors have you heard? Where do you think this came from?
3. Who have you heard these from?
4. How do you feel about what you’ve heard?
5. ***[If untrue]*** What do you think is the best way to address these ideas/rumors?
   - 1. Who should address these?
     2. Is there anything the clinic has done or could do to address community rumors or stigma?

***Theme 7: As we finish [for ALL]***

1. As we come to the end of our time today, I’d like you to think about your sister, or a close friend and tell me whether you would recommend that she use PrEP?
2. Why?
   - 1. What about this person makes you think that they should/should not use PrEP?
     2. What is it about PrEP that you would/would not recommend?
3. Do you have anything else you’d like to tell me about related to your experience with PrEP or PrEP services?

**In-depth Interview Guide – Healthcare Providers**

**{FOR ALL} Theme 1: Background Info**

1. Can you tell me what you mainly do at [site]? In what service area (e.g., ANC, PHC, ART)?
2. Do you see many adolescent girls and young women in your work?
3. **[If not already discussed]** How, if at all, are you involved at all in PrEP service delivery?

**{FOR ALL} Theme 2: HIV Prevention for Adolescent Girls and Young Women**

1. For the adolescent girls and young women you see, what do you communicate about HIV prevention (i.e., they should do ______, they should know________)?
   1. If this different for some adolescent girls and young women versus others (e.g., younger v. older, married v. not married)? Different than what you would say adults?
   2. How concerned about HIV are adolescent girls and young women? How do these compare to their other concerns (i.e., about getting pregnant or others)?
2. What methods of prevention do you promote to adolescent girls and young women? Why?

**{FOR ALL} Theme 3: PrEP and PrEP Access**

1. How much do you already know about PrEP?
   1. Where and how did you find out? *(Note: don’t care about this experience)*
2. What do you think about PrEP?
   1. How confident are you that PrEP can prevent HIV infection?
   2. What concerns do you have about PrEP?
3. What do you think about providing PrEP to adolescent girls and young women? Why?
   1. What have you heard others at [site] say about AGYW taking PrEP?
4. Where do you think PrEP services for adolescent girls and young women should be provided? Why is that?
5. What would be/are the advantages of delivering PrEP for AGYW in a facility? Disadvantages?
6. What would be/are the advantages of delivering in the community? Disadvantages?
7. Within a facility, where should PrEP services for adolescent girls and young women be provided? (e.g., FP, ANC/PNC) Why? Are they provided like this now? If no, why not?
8. **[if DREAMS]** How well would it/did it work to refer clients to facilities for follow-up? Why? What could help it to work better?
9. **[if facility]** How well would it/did it work to receive clients who were started on PrEP in the community? Why? What could help it to work better?

**{FOR ALL} Theme 4: Feasibilty and Acceptability of PrEP Service Delivery**

1. Before you started **[or if you never started]**, what did you think about delivering PrEP?
   1. Why did you/do you want to or not want to deliver PrEP services?
   2. What were your/are your concerns? What, if any, concerns did you have/do you have about delivering PrEP to adolescent girls and young women?
   3. How confident were you at that time/are you about delivering PrEP services?
   4. **[If delivers PrEP services now]** Do you still feel this way now? Why or why not?
2. How do you think it was decided to start PrEP service delivery at [site]? Was it something that came from the Ministry or did it come from somewhere external?
3. Do you think management and staff at [site] are in favor of delivering PrEP? Why?
4. How much is delivering PrEP a priority compared to other services? Why?
5. Within PrEP service delivery, how much focus has been put on delivering PrEP to adolescent girls and young women specifically?
6. Do you think the right people are involved in PrEP service delivery at [site]? Why? Who else should be involved? Why/what would be gained from their involvement? What, if anything, has prevented them from being involved?
7. Who has helped to lead or motivate you or other staff to provide PrEP services for adolescent girls and young women? How so/what do they do?
8. From what you know, how complicated do you think it is to deliver PrEP?
9. Are there parts of PrEP service delivery that aren’t practical? Which ones? Why?
10. How well do you think PrEP fits with the other services you provide at [site]?
11. Do you think [site] has the resources needed to deliver PrEP services? What is missing? (probe on PrEP commodity)

**{For people who deliver PrEP-related services}
Theme 5: Personal Experiences Delivering PrEP**

1. Next, I’ll ask you a few questions about what PrEP delivery looks like at [site].
2. Who provides PrEP education? (Probe for multiple cadres) What do they do? Do you use any materials and if so have you had any challenges (e.g., availability)?
3. Who provides PrEP counseling? (Probe for multiple cadres) What topics do they cover? Are these topics different at the initiation visit versus follow-up visit?
4. **[if nurse]** How do you identify who to or not to offer PrEP? (i.e., risk assessment)
5. What is difficult about [delivering PrEP services] for you at [site/community]? How so?
   1. What could be done to help make this less difficult?
6. What makes it easier for you to [*deliver PrEP services – customize to their role*]?
7. Do you use any materials/job aids? How helpful are they? What else would be helpful?
8. Who do you go to when you have questions or want more information about PrEP or PrEP service delivery? Is this helpful? Why or why not?
9. How do others outside of [site] help you with implementing PrEP? What do they do?
10. Do you ever receive any feedback (e.g., verbal or data) about how well service delivery is going? How do you use that information?
11. What do you think helps adolescent girls and young women decide whether or not to start PrEP? Why do you think [this/these things] helps? (Probe about flyers if not mentioned)
12. Are [these things] currently done by your or others at [site]?
    - 1. If yes, how well do [these things] work? Why do you say that?
      2. If no, why not? Would it be feasible to do [these things]? Why or why not?
13. What do you think helps adolescent girls and young women continue taking PrEP over time? Why do you think [this/these things] helps?
14. Are [these things] currently done by your or others at [site]?
    - 1. If yes, how well do [these things] work? Why do you say that?
      2. If no, why not? Would it be feasible to do [these things]? Why or why not?
15. We see a large number of adolescent girls and young women who never come back for PrEP after initiation. Why do you think this happens? What would help? Why or why not?
16. How have adolescent girls and young women responded to PrEP services at [site]? Why?
17. What barriers do adolescent girls and young women face in accessing and continuing PrEP services at [site]?
18. **[If DREAMS]** What barriers do adolescent girls and young women face in accessing and continuing PrEP services at MoHSS health facilities?

**{For ALL} Theme 6: Wrap-up**

1. Is there anything else we should know about PrEP service delivery for adolescent girls and young women? About PrEP service delivery in general?
