# Supplementary material for: Improving HIV pre-exposure prophylaxis persistence among adolescent girls and young women: Insights from a mixed-methods evaluation of community, hybrid, and facility service delivery models in Namibia
Source: Front Reprod Health. 2022 Dec 5;4:1048702. doi: 10.3389/frph.2022.1048702 (PMC9760915; doi:10.3389/frph.2022.1048702)
Supplement: Supplementary file 3 [file Table3.docx]

**Table S3.** Additional quotations illustrative of mechanisms supportive of and detractive from community and hybrid models achieving improved pre-exposure prophylaxis persistence among adolescent girls and young women

| Theme | Illustrative quotations |
| --- | --- |
| *Supportive mechanisms* | |
| Convenience and simplicity | *[At] the clinic, I have to pay taxi money. At home, in the community, it’s safe and it’s simple. I can just go there by foot, go get my PrEP and I go back. —AGYW IDI 81, age 20* |
|  | *They just gave me a date, then they told me, if I don’t get the time to go there, then they will come to me… I wanted to go there but I forgot that day, I really forgot until they called me like, ‘Ah your date is today.’ And I was like, ‘Aww! I forgot, where are you?’ and they were like, ‘We have already left, now we are just coming for you.’ So they came straight… and then they gave me three bottles… I had another follow-up and they still kept coming to my place actually… Ok, honestly, I don’t keep track of my day, they do… It has helped me because I would have been missing out. —AGYW IDI 83, age 20* |
| Convenience and simplicity – challenges | *We found this other lady calling the [community-based] nurses, ‘tell them that there are two girls who came for follow-up.’ Then the [nurses] said, ‘we are coming, they must just give us 15 minutes.’ And we have to go back to school. We stayed and they weren’t coming. 15 minutes went by… we went back to school. —AGYW IDI 74, age 22* |
|  | *Yes, there was this one, there was this day that I was travelling to my boyfriend in Okahandja. So, while I was travelling, when I was packing my things, I forgot to pack my PrEP. So, I just went to Okahandja. And it’s like, and when I went there it is like, how can I say like? It is like I just totally forgot about PrEP. It was for a week. And then, um, when I went to church and came back, I saw this woman. And she was having a PrEP. And I was like, my PrEP! I forgot my PrEP. Oh my God. I can’t believe it. I forgot my PrEP. And I was so nervous that I was telling my boyfriend I want to go back now and go get my PrEP. And he was like why should you go back? You can just get other pills. No, I should go back and get my PrEP. —AGYW IDI 92, age 17* |
| Convenience and simplicity – implementation | *Mostly I get fetched from school by the nurses, they come with a car looking for us… Because they asked us, telling us that, ‘if you are treated like that at the clinic, you cannot keep on going there. Because it’s like those people they will end up discouraging you, talking about useless things.’ Then they asked, ‘do you want us to come and get you from school?’ Because they wanted to give them to me from home, but my phone is mostly off. They called me but they couldn’t reach me, that’s why they come and look for us at school. — AGYW IDI 74, age 22* |
|  | *There is a lot of phone calls. You have to call, and DREAMS is providing credit for that. No problem. And there is transport without problem. When we have to travel, they take you to the girl. Anywhere. —HCP IDI 22, health assistant* |
|  | *At times, people come with different ideas, like why don’t you just call girls to come into the safe space where they were initiated, and then we tried to follow them up in that same safe space. We tried that; it didn’t work. You call them up, ‘okay we will bring transport, just come to the safe space.’ Out of 4, you just find maybe 2 or only one comes. The rest, ‘no we couldn’t come, I have a baby at home, no I was at school, I couldn’t come.’ You know they will give different excuses. And then you would also get some adolescent girls where you go into the community, they will tell you, ‘okay, let’s meet where, where.’ When you reach that point, you start calling and either they don’t answer the phone, they switched off the phone, or they block your number. So, it actually takes a lot of patience. —HCP IDI 25, nurse* |
| *Supportive mechanisms* | |
| Social connectedness with providers – antecedents to connectedness | *So I always feel like it’s part of being youth friendly. Trying to build relationships with your clients, trying to understand that okay, I am dealing with this age group, how does this age group behave.” —HCP IDI 25, nurse* |
|  | *Because with us, you have to build trust first before we go through with the conversation. The trust is already there, cause whether we are 3 or 4 we have to talk about confidentiality and building trust with the client before we go start everything. So it cannot give us a problem. And we mostly use to tell them that what we discuss here, whether we use to talk to the mother, we don’t have the right to talk about your PrEP, your result, your boyfriend’s status or whatever. To your mother since you don’t want. —HCP IDI 24, health assistant* |
|  | *And then the follow-up phone calls I think those are very important just to find out, you know at times, like even me as an individual, I go visit you at a clinic or a hospital if I wasn’t feeling well and then I get this follow-up call maybe a week later or so. Since you came last time I figure, it’s just, you know it makes you feel good. It also makes you feel like oh, those people really caring, unlike if you just giving me medication and I am done with you. —HCP IDI 25, nurse* |
|  | *No, she was just open to us, she would just like tell us everything, she will not hide anything from us. —AGYW IDI 76, age 18* |
|  | *I was feeling more comfortable. They were also girls like me (laughs). Just like I’m talking to my sister or to my mom. Just the same thing.” —AGYW IDI 81, age 20* |
| Social connectedness with providers – social support | *That it can damage your liver, yeah, it’s what [the person] said, it can damage your liver and everything. So the moment I hear that I can damage your liver, I felt like no, I will just drop this pill. And I told her, ‘I want to drop this pill.’ And she was like, ‘if you want to drop this pill then you have to talk to your nurses.’ When I met my nurses the next day, when they came to get us from school, we told them about that. Even the way the lady was telling us, I told them everything. Then they gave me some counselling, ‘we are not forcing you because force is a crime, it is your own decision, it is you who decided to take this pill. If you feel like you want to drop it, it’s on you and if you feel like continuing, it’s on your own.’… That thing that I had that I will drop it, it completely got out of my head and they asked me, ‘are you dropping it or are you continuing?’ And I said, ‘I’m continuing because it’s my life.’… So I just continued. — AGYW IDI 74, age 22* |
|  | *For the person to continue [taking] pills, she needs to be given enough information for her to understand that PrEP pills do what … and what it does in a person for this person to have motivation to [take PrEP]. Because if a person is only told that the pill prevents diseases while not knowing the whole information, then now she won’t be motivated to continue like that. —AGYW IDI 79, age 23* |
|  | *“PrEP is taken every day. The neighbors or people from the house, they keep thinking that maybe this person is infected with the virus, or why [else] does she have to be taking pills. I have been thinking that if I start taking them, what would people be thinking about me? I was just like, maybe I should just take them, maybe they are going to think that I have the virus, like that. It really got to me and it brought me to the point of not getting PrEP but when I went to ask [the community-based providers], asking for more information. I realized that in life if you are following what [other] people are saying, you will be left behind. It’s better that I take PrEP, whoever is going to talk or do what. Then I can tell them about PrEP, that it has to do with what, until they hear about it well those that reach out to me. —AGYW IDI 80, age 22* |
|  | *Yeah, I had the intention that if I am going to restart, I’ll just go to the clinic and really ask for information. I’ll go to the nurse and really ask her for information about how they are taken, in what way can I take these pills. And if perhaps I get ill again, what can I do? Can I continue, or is it maybe only because it is in the beginning when I start taking them? Something like that. That is the information I wanted to ask, to go ask so that I can continue. Okay I took them, can I stop them or is it a problem again to me if I stop them? —AGYW IDI 70, age 23* |
| *Supportive mechanisms* | |
| Social connectedness with peers – antecedents of connectedness | *We did [PrEP education] like with everyone that was present…Everyone was given a chance to answer, they asked questions, just like that… It was fine because we are all girls. We experience everything, we don’t need to be shy of each other. —AGYW IDI 84, age 24* |
|  | *Okay so, usually we [DREAMS counsellors] use the counselling [approach] where you try to find out how much the client knows a about a certain topic that you are giving out. So, even with the school sessions that we are giving we tend to go even with power point presentations where you try to ask then what do you know about HIV. We hear different view about what the learners are saying HIV is. How it’s spread and all. Then later on we can give the presentation trying to explain [more]. It also helps clear out any myths that the learners have and after that we can even give a group exercise where they work together explaining what they think. Just give a topic or a question that they can answer as a group and at times we use to have some questions and answer like shifts where they can actually respond is this statement true or false about HIV. And then when a client comes for health services it can be done in a group like while they are waiting for services, we can talk about it like as they are sitting in groups. —HCP IDI 25, nurse* |
| Social connectedness with peers – social support | *I would change, when the people are going for follow-ups, to get PrEP, I think that there should be a time where we sit together and talk as ladies. All of us that are taking PrEP, that are coming to get PrEP, we should sit and talk as ladies. We talk about PrEP, about how you feel taking it, and how it has been affecting you. Or if you have a problem taking PrEP we can share, you can talk to us, give your advice. So that we can share our feelings. —AGYW IDI 92, age 17* |
|  | *I remember just yesterday. There was someone on PrEP. I asked her, ‘are you still taking your medicine?’ Then she was like, “no, I don’t take them anymore, I stopped.” I was like, ‘why did you stop?’ She said, ‘no, my parents said I should stop.’ Those kinds of things. Then I told her that, ‘but when you took it, did you tell them [about PrEP]?’ She said, ‘Yes I told them.’ Then I told her more about PrEP and she said she will check if she can go back on it. —AGYW IDI 74, age 22* |
| Social connectedness with peers – risk of stigma | *Yes. There was a time when I stopped PrEP for a while. Because of the um, how can I say? Because of, I would have the self-esteem. Because of my friends judging me… I would always drink the tablets at work. But we would always sit and watch movies together. I would always carry the tablets in my handbag. And every time I would drink the tablet, around 20h00. They would say, ‘what are you sick, are you sick with HIV?’ and all that. And they would tell me, ‘no leave that tablet or else you would not hang with us. Don’t take the tablet. That tablet is not okay. And it does not suit you.’ And all that. Yes. I had low self-esteem. I did not take PrEP; I think it was for a month. I had low self-esteem. I did not have confidence in myself. I would, how can I say? I would choose my friends over PrEP. —AGYW IDI 92, age 17* |
|  | *I was worried like because we are friends. Some of them they are saying, uh-huh those things are for those who are HIV positive and some of them they are saying, umm you will get HIV you don’t know that they are made with what. Just like that. The pills are made with what, maybe you will be infected, something like that. —AGYW IDI 70, age 23* |
| *Detractive mechanisms* | |
| Apprehension over unfamiliar services and providers | *Actually, they wrote us that, cause I live in (location), so the nearest place I could get PrEP was (high school). But actually, I didn’t go there because the nurses will call when it’s your follow-up day and they will come to your house. Yes, I like it cause I don’t like going to other nurses again that I am not used to. So, I prefer being with them that I am used to now. —AGYW IDI 94, age 18* |
|  | *Sometimes you go somewhere, but it’s not where you are supposed to go. Then they will send you back, saying, ‘you’re first supposed to start at the testing’… then you go on the scale, from the scale you go to the doctor, the doctor sometimes will send you to go withdraw blood here. —AGYW IDI 75, age 24* |
|  | *First it was, it was pretty challenging. Cause the facilitator that was doing the project with us at the church was not there [at the clinic]. So, when I went there [to the clinic], I was just looking for the DREAMS program, anyone. —AGYW IDI 97, age 20* |
|  | *So, for the first [follow-up] visit, the nurse already knows that I’m taking PrEP and I have PrEP with me. So why would she ask me again why I’m taking PrEP. Because I already told other people I’m taking PrEP and the reason why I’m taking PrEP.” —AGYW IDI 72, age 17* |
|  | *[Healthcare providers in the facility] are saying that it’s [PrEP] only for people that are having partners [with] HIV, sometimes they might not give you [PrEP]. —AGYW IDI 78, age 24* |
