# Supplementary material for: Improving HIV pre-exposure prophylaxis persistence among adolescent girls and young women: Insights from a mixed-methods evaluation of community, hybrid, and facility service delivery models in Namibia
Source: Front Reprod Health. 2022 Dec 5;4:1048702. doi: 10.3389/frph.2022.1048702 (PMC9760915; doi:10.3389/frph.2022.1048702)
Supplement: Supplementary file 4 [file Table4.docx]

**Table S4**: Synthesis of potential mechanisms that explain how and why the community-concierge and hybrid community-clinic service delivery models achieved (or not) improved PrEP persistence among AGYW in Khomas region, Namibia

| **Mechanism title** | **Service delivery component(s)** | **Context** | **Mechanism** | **Outcome(s)** | **Considerations** |
| --- | --- | --- | --- | --- | --- |
| *Supportive mechanisms* | | | | | |
| Convenience and simplicity | Individualized approach to the delivery of PrEP refills  Client could select their preferred community-based PrEP refill location at initiation  Changes to the location, day, and time of the refill could be made in two ways:  1) Provider-initiated phone call with the AGYW approx. one week before the scheduled refill  2) Client-initiated SMS, WhatsApp, or phone call to provider | *Component of the community-concierge model only*  AGYW have limited time and resources and many competing priorities  AGYW schedules frequently changing | Individualization maximized convenience and simplicity of obtaining refills and accommodated real-time constraints and preferences. | Reduced financial, time, and opportunity costs for AGYW in obtaining PrEP refills | Did not work for mobile AGYW who traveled outside of the service delivery catchment area  Did not work for AGYW without reliable and consistent mobile phone number  Implementation is resource-intensive (e.g., time, phone credit, transportation)  Concerns and challenges about maintaining privacy when delivering services in community-based locations |
| Social connectedness with providers | Providers intentionally employed strategies to establish positive relationships with AGYW– non-judgmental, explicit assurances of confidentiality, friendly interpersonal communication, wellbeing check-ins by phone shortly after initiation, sharing personal experiences with HIV and PrEP. | AGYW anticipate and experience provider mistreatment and limited provider continuity at facilities  Community-based providers were predominately young and female | Positive relationships enabled AGYW to experience social connectedness with their providers allowing for open communication and increasing AGYW’s confidence in the availability and appropriateness of the social support provided.  Emotional, informational, and appraisal support from providers enabled AGYW to make informed decisions about their PrEP use, respond to challenges, and to seek further social support from family, partners, or others. | Increased emotional, informational, and practical support  Increased self-efficacy and self-management of PrEP use | AGYW may delay obtaining PrEP or chose less convenient locations in order to see a known and trusted provider |
| *Supportive mechanisms* | | | | | |
| Social connectedness with peers | PrEP-related services were layered onto HIV prevention programming delivered to small groups of similarly aged AGYW who regularly met over several weeks. AGYW received group-based PrEP education (delivered by a peer mentor) and health services (incl. PrEP) were delivered to these groups during drop-in days (though clinical services were delivered one-to-one with a provider). AGYW regularly met before and after receiving health services. | Peers play key role in AGYW social lives and exert a strong influence on decision making | Consistent interactions, a desire to connect with peers, and sharing similar experiences and characteristics fostered connectedness between AGYW and their same sex/age peers building social networks and systems of social support: role models influencing decisions to start and persist with PrEP; offer practical support incl. techniques to cope with challenges and experiential knowledge; and provide mutual adherence support | Increased self-management of PrEP use | AGYW may engage peers in other social networks and encounter stigma and discouragement |
| *Detractive mechanisms* | | | | | |
| Apprehension over unfamiliar PrEP services and providers | AGYW who initiated PrEP in the community were referred to their preferred government health facility for PrEP refills and follow-up to reduce resource and provider burden and support sustainability.  AGYW were provided with a stamped paper confirming their community-based PrEP initiation (as community providers could not give AGYW MoHSS health passports) to facilitate referral and PrEP refill/follow-up at facilities. | Positive provider and service experiences with community-based PrEP services  Community-based PrEP was an externally funded program.  Limited collaboration and communication between community and facility services  Substantially more AGYW initiating PrEP in community than facilities | Requiring AGYW to access refill/follow-up services at facilities introduced fear and anxiety among AGYW about how to access services, what services delivery would be like, how they would be treated as well as reintroduced access barriers associated with facility-based services. | Fewer AGYW seeking PrEP refills  Increased time spent navigating unfamiliar facility-based PrEP services  Some AGYW encountered unhelpful, discouraging and/or uninformed providers at government health facilities. | AGYW may lose or forget referral paper which may make it more difficult for them to obtain PrEP at referral location  Limited collaboration and communication between community and facility providers can introduce mistrust and concerns about service quality which can affect how AGYW who initiated PrEP in the community are treated at facilities or other service locations. |
